# Detection of Genetic Diversity in *Campylobacter jejuni* Isolated from a Commercial Turkey Flock Using *flaA* Typing, MLST Analysis and Microarray Assay

**DOI:** 10.1371/journal.pone.0051582

**Published:** 2013-02-20

**Authors:** Hosny El-Adawy, Helmut Hotzel, Herbert Tomaso, Heinrich Neubauer, Eduardo N. Taboada, Ralf Ehricht, Hafez M. Hafez

**Affiliations:** 1 Friedrich-Loeffler-Institut, Institute of Bacterial Infections and Zoonoses, Jena, Germany; 2 Institute for Poultry Diseases, Free University Berlin, Berlin, Germany; 3 Department of Poultry Diseases, Faculty of Veterinary Medicine, Kafrelsheikh University, Kafr El-Sheikh, Egypt; 4 Laboratory for Foodborne Zoonoses, Public Health Agency of Canada, Lethbridge, Canada; 5 Alere Technologies GmbH, Jena, Germany; University College Dublin, Ireland

## Abstract

*Campylobacter* is genetically highly diverse and undergoes frequent intraspecific recombination. Turkeys have been identified as an important reservoir for *Campylobacter jejuni* which is of public health significance. The assessment of the genetic diversity among *Campylobacter* population is critical for our understanding of the epidemiology of this bacterium. The genetic profiles were different according to the molecular typing methods used. The performance of established *flaA* genotyping, multilocus sequencing typing (MLST) and DNA microarray assay based on the ArrayTube™ technology was evaluated using 14 *Campylobacter jejuni* isolated from a commercial turkey flock. The *flaA* typing was performed using PCR-RFLP with restriction enzymes *Sau3AI, AluI*, a ‘composite’ *flaA* analysis of *AluI* and *Sau3AI* and *DdeI*. The 14 isolates were differentiated into 3, 5, 7 and 9 genotypes, respectively. Entire *flaA* gene and short variable region (SVR) sequences were analysed. Sequencing of the entire *flaA* provided 11 different genotypes. *flaA*-SVR sequence analysis detected 8 *flaA* alleles and 4 *flaA* peptides. One new *flaA* allele type (528) was identified. MLST analysis represented 10 different sequence types (STs) and 5 clonal complexes (CCs). The microarray assay recognised 14 different genotypes. The discriminatory indices were 0.560, 0.802, 0.857, and 0.912 for *flaA*-RFLP depending on the used enzymes, 0.890 for *flaA*-SVR, 0.967 for entire *flaA* sequencing, 0.945 for MLST and 1.00 for the DNA microarray assay. The *flaA* gene was genetically stable over 20 passages on blood agar. In conclusion, the different typing tools demonstrated a high level of genetic heterogeneity of *Campylobacter jejuni* in a turkey flock, indicating that a single flock can be infected by multiple genotypes within one rearing cycle. DNA microarray-based assays had the highest discriminatory power when compared with other genotyping tools.

## Introduction

*Campylobacter* is recognized as the leading cause of bacterial gastroenteritis in Europe and a significant public health concern worldwide. Poultry and poultry products remain the most important source of food-borne human campylobacteriosis [1], [2]. Genetic diversity among thermophilic *Campylobacter* spp. may enable survival of these bacteria in the environment by means of variation in strain virulence [3]. Advanced molecular typing tools have improved our understanding of the epidemiology of bacterial food-borne pathogens. Monitoring of poultry flocks has shown that some are infected with only one genotype of *Campylobacter* spp., while more than one genotype has been detected in others [4], [5]. Different strains in individual flocks may be replaced or displaced by others during the rearing cycle [6], [7]. Some clones of *C. jejuni* remain genetically stable in completely different environments over long periods of time and considerable geographical distances. Moreover, the human isolates remained stable for almost 20 years under laboratory conditions [8].

There are various methods used for typing *C. jejuni* [9]. It has been suggested that the sensitivity of the *flaA* gene locus to spontaneous genetic change is a limiting factor in its use in long-term epidemiological studies, but is suitable for the initial grouping of isolates in surveillance situations [10]. The *flaA* gene of *Campylobacter* species serves as an epidemiological marker, as it shows extensive sequence heterogeneity [9]. The *flaA* typing in *C. jejuni* is a commonly used, rapid and easy method for genotyping with an acceptable discriminatory power [6], [11], [12]. It has been shown that PCR-RFLP of *flaA* amplicons was suitable for discriminating *C. jejuni* isolates by generating DNA banding pattern [13]. Different restriction enzymes can be used, and combining the enzyme patterns (composite analysis) has been shown to result in an increased degree of discrimination [9], [14]. Sequencing of the entire *flaA* gene is a highly reproducible method, allowing precise and simple worldwide comparison of isolates [15]. The entire coding sequence of *flaA* gene (1,764 nucleotides) of *C. jejuni* contains two regions of high variability, one region from approximately base positions 700 to 1,450 and a short variable region (SVR) from base positions 450 to 600 [12], [16].

Multilocus sequence typing (MLST) is suitable for the investigation of diverse bacterial populations which have weakly clonal population structures [17], [18]. The MLST scheme displays high portability and great facility for inter-laboratory comparisons, which has contributed to a greater understanding of the population structure and global epidemiology of *C. jejuni* and related organisms [19].

*C. jejuni* has approximately 1300 core and house-keeping genes that encode functions required for survival, as determined by comparative genomic sequencing [20] and by microarray-based comparative genomic hybridization analysis [21], [22]. Whole genome DNA microarrays are used to investigate the genomic dynamics through determination the presence or absence of thousands of genes in a single hybridization experiment. They are suitable for rapid and accurate simultaneous differentiation among thermophilic campylobacters [23], [24]. The ArrayTube™ (AT^TM^) system is a less expensive platform and characterizes *C. jejuni* isolates by specific hybridization patterns of selected gene loci. The advantage of the AT^TM^ system is enzyme-catalysed precipitation staining rather than fluorescence detection of positive hybridization signals; moreover, the signal intensities are measured by a simple transmission technique [25], [26].

The discriminatory power of the different genotyping methods was determined by the measure of resolution that could be achieved by the respective methods. It is given as a numerical value, which can be used for simple comparison between methods [27].

The objective of this study was to determine the genetic diversity among *C. jejuni* isolates recovered from a single turkey flock during the production cycle. Sampling and cultivation of *C. jejuni* isolates were carried out from the beginning of flock colonization until slaughter. The *C. jejuni* heterogeneity was investigated using different genotyping tools such as *flaA* typing, MLST and microarray analysis. The performance of the different genotyping methods was evaluated based on their discriminatory power, costs per isolate, ease of handling, and time-to-result.

## Materials and Methods

### Bacterial strains and growth conditions

Fourteen *C. jejuni* isolated from cecal content of a turkey flock reared in a single farm. The flock was sampled at several dates (Table 1) from the beginning until the end of the production cycle. The isolation was carried out according to ISO 10272 [28]. Briefly, 1 g of fecal sample was inoculated into 9 ml of Bolton selective enrichment broth (Oxoid Deutschland GmbH, Wesel, Germany) and incubated at 42°C for 24 h under microaerophilic conditions (5% O_2_, 10% CO_2_, 85% N_2_) (Jenny medical-Trilab, Schütt Labortechnik, Göttingen, Germany). A loop of broth was plated onto modified charcoal cefoperazone deoxycholate agar (mCCDA) (Oxoid Deutschland GmbH) and Brilliance CampyCount Agar (Oxoid Deutschland GmbH). Incubation was done at 37°C for 6 h followed by incubation at 42°C for 18–36 h under microaerophilic conditions. Suspected colonies were subcultured on Mueller Hinton (MH) blood agar (10% citrated bovine blood). Bacterial cultures were identified phenotypically by Gram staining and by the API 20E system (bioMerieux Deutschland GmbH, Nürtingen, Germany).

**Table 1:** Restriction profiles of *flaA* typing, *flaA*-SVR alleles (321 nucleotides) and *flaA*-SVR peptides (107 peptides) of 14 *C. jejuni* isolates, their accession numbers and the date of isolation.

Isolates	*Sau3AI* (A–C)	*AluI* (1–5)	*AluI*/*Sau3AI* (I–VII)	*DdeI* (a–i)	*flaA*-SVR alleles	*flaA*-SVR peptides	Accession No.	Date of isolation
CS0048	B	4	I	a	105	1	JQ991581	04-05-10
CS0052	A	1	II	b	515	1	JQ991582	17-06-10
CS0073	A	4	IV	c	18	20	JQ991583	07-07-10
CS0074	A	3	III	d	1124	1	JQ991584	07-07-10
CS0075	B	4	V	e	34	1	JQ991585	07-07-10
CS0076	A	3	III	d	1124	1	JQ991586	07-07-10
CS0077	A	3	III	d	1124	1	JQ991587	07-07-10
CS0078	C	2	VI	f	359	9	JQ991588	07-07-10
CS0079	B	4	V	g	34	1	JQ991589	07-07-10
CS0080	A	3	III	d	1124	1	JQ991590	07-07-10
CS0081	A	1	II	b	515	1	JQ991591	07-07-10
CS0082	A	1	II	h	515	1	JQ991592	21-07-10
CS0083	A	1	II	h	528	1	JQ991593	21-07-10
CS0084	C	5	VII	i	16	12	JQ991594	21-07-10

### DNA Extraction

Genomic DNA was extracted from a 48 h bacterial culture on MH blood agar plates using High Pure PCR Template Preparation Kits (Roche Diagnostics GmbH, Mannheim, Germany) according to the manufacturer's instructions. The DNA was eluted in 200 µl elution buffer. DNA was quantified spectrophotometrically using a Nanodrop® ND-1000 (Fisher Scientific GmbH, Schwerte, Germany).

### Species confirmation and *flaA*-RFLP assays

The identified isolates were confirmed as *C. jejuni* by using a multiplex PCR (mPCR) assay as described previously [29]. For *flaA*-RFLP analysis extracted DNA was amplified, as described elsewhere [13], using modified primers with nucleotide sequences as given in Table 2. Amplification conditions were: initial denaturation for 60 s at 94°C followed by 35 cycles each consisting of 15 s at 94°C, 60 s at 45°C, 120 s at 72°C and a final extension step of 300 s at 72°C. The *flaA* amplicon was digested for 18 h at 37°C with *AluI* (Jena Bioscience GmbH, Jena, Germany), *DdeI* (Roche Diagnostics GmbH), *Sau3AI* (Jena Bioscience GmbH), and a mixture of *Sau3AI* and *AluI* enzymes using the incubation buffer recommended by the manufacturers. The DNA segments were separated using 2.5% agarose gels (Starlab GmbH, Hamburg, Germany) in TBE buffer at 200 V for 1 h, stained with ethidium bromide and visualized under UV light. Documentation was done using a Bio Imaging System (Syngene, Cambridge, UK).

**Table 2:** Primers used for *flaA* typing and MLST of *C. jejuni* isolates.

Primer	Sequence	Gene	Aim	Amplicon bp
flaA1-Wob	5′-GGATTTCGTATTAACA-3′	*flaA*	Amplification	∼1,700
fla 2-Wob	5′-CTGTARYAATCTTAAAACATTTTG-3′	*flaA*	Amplification	∼1,700
flaA-S-1	5′-GCAGATGATGCTTCAGGG-3′	*flaA*	Sequencing	
flaA-S-2	5′-CTGCTATCGCCATCCCTG-3′	*flaA*	Sequencing	
flaA-S-3	5′-AAATCAAGTYACATCRAC-3′	*flaA*	Sequencing	
flaA-S-4	5′-AGAGTARTTTGCACTCTC-3′	*flaA*	Sequencing	
flaA-S-5	5′-GATAAGGCTATGGATGAGC-3′	*flaA*	Sequencing	
flaA-S-6	5′-GCTCTGATTTGATCAAG-3′	*flaA*	Sequencing	
flaA-S-8	5′-AAGTGTGGTAACACCTGC-3′	*flaA*	Sequencing	
flaA-S-9	5′-CCYACWGAAWAWCCYGAACC-3′	*flaA*	Sequencing	
flaA-S-10	5′-TCAAGAATTTCAAATCGG-3′	*flaA*	Sequencing	
flaA-S-11	5′-AAAKCCCATAGCATCRGC-3′	*flaA*	Sequencing	
flaA-S-12	5′-TTACTCTTAAAAACTAC-3′	*flaA*	Sequencing	
flaA-S-13	5′-CCATCATTTTTAACTAAA-3′	*flaA*	Sequencing	
asp-A9	5′-AGTACTAATGATGCTTATCC-3′	*aspA*	Amplification	899
asp-A10	5′-ATTTCATCAATTTGTTCTTTGC-3′	*aspA*	Amplification	899
asp-S3	5′-CCAACTGCAAGATGCTGTACC-3′	*aspA*	Sequencing	
asp-S6	5′-TTAATTTGCGGTAATACCATC-3′	*aspA*	Sequencing	
gln-A1	5′-TAGGAACTTGGCATCATATTACC-3′	*glnA*	Amplification	1,262
gln-A2	5′-TTGGACGAGCTTCTACTGGC-3′	*glnA*	Amplification	1,262
gln-S3	5′-CATGCAATCAATGAAGAAAC-3′	*glnA*	Sequencing	
gln-S6	5′-TTCCATAAGCTCATATGAAC-3′	*glnA*	Sequencing	
glt-A1	5′-GGGCTTGACTTCTACAGCTACTTG-3′	*gltA*	Amplification	1,012
glt-A2	5′-CCAAATAAAGTTGTCTTGGACGG-3′	*gltA*	Amplification	1,012
glt-S1	5′-GTGGCTATCCTATAGAGTGGC-3′	*gltA*	Sequencing	
glt-S6	5′-CCAAAGCGCACCAATACCTG-3′	*gltA*	Sequencing	
gly-A1	5′-GAGTTAGAGCGTCAATGTGAAGG-3′	*glyA*	Amplification	816
gly-A2	5′-AAACCTCTGGCAGTAAGGGC-3′	*glyA*	Amplification	816
gly-S3	5′-AGCTAATCAAGGTGTTTATGCGG-3′	*glyA*	Sequencing	
gly-S4	5′-AGGTGATTATCCGTTCCATCGC-3′	*glyA*	Sequencing	
pgm-A7	5′-TACTAATAATATCTTAGTAGG-3′	*pgm*	Amplification	1,150
pgm-A8	5′-CACAACATTTTTCATTTCTTTTTC-3′	*pgm*	Amplification	1,150
pgm-S2	5′-TCCAGAATAGCGAAATAAGG-3′	*pgm*	Sequencing	
pgm-S5	5′-GGTTTTAGATGTGGCTCATG-3′	*pgm*	Sequencing	
tkt-A3	5′-GCAAACTCAGGACACCCAGG-3′	*tkt*	Amplification	1,102
tkt-A6	5′-AAAGCATTGTTAATGGCTGC-3′	*tkt*	Amplification	1,102
tkt-S4	5′-ACTTCTTCACCCAAAGGTGCG-3′	*tkt*	Sequencing	
tkt-S5	5′-GCTTAGCAGATATTTTAAGTG-3′	*tkt*	Sequencing	
unc-A7	5′-ATGGACTTAAGAATATTATGGC-3′	*uncA*	Amplification	1,120
unc-A2	5′-GCTAAGCGGAGAATAAGGTGG-3′	*uncA*	Amplification	1,120
unc-S4	5′-TGCCTCATCTAAATCACTAGC-3′	*uncA*	Sequencing	
unc-S5	5′-TGTTGCAATTGGTCAAAAGC-3′	*uncA*	Sequencing	

primers created for this study.

primers according to [18].

### Analysis of *flaA*-RFLP results

TIF images of the restriction profiles for *flaA*-RFLP were incorporated for analysis into BioNumerics V. 4.50 (Applied Maths, Austin, TX, USA). Pair comparisons and cluster analysis were made using the Dice correlation coefficient and the unweighted pair group mathematical average (UPGMA) clustering algorithm. The optimization and position tolerance for band analysis were set at 4%, and a cut-off of 90% was used for the determination of the different restriction patterns for *flaA*-RFLP.

### *In vitro* stress model (genetic stability test)

Seven *C. jejuni* isolates derived from single colonies (CS0048, CS0052, CS0073, CS0077, CS0078, CS0079, and CS0084) with different restriction profiles of the *flaA* genes digested with *DdeI* were selected for stress test. Isolates were stored in cryovials (Mast Diagnostica Laboratoriums-Präparate GmbH, Reinfeld, Germany) at −80°C after the first isolation, and none were subcultured or cloned prior to analysis. The isolates were grown on MH blood agar plates (Oxoid Deutschland GmbH) supplemented with 10% citrated bovine blood and incubated for 24 h at 42°C under microaerophilic conditions (5% O_2_, 10% CO_2_, 85% N_2_) (Jenny medical-Trilab, Schütt Labortechnik). Isolates were then subcultured 20 times on MH blood agar for 48 h at 42°C in a microaerophilic atmosphere. After the 4^th^, 8^th^, 12^th^, 16^th^ and 20^th^ passage, chromosomal DNA was extracted from selected isolates of these passages and subtyped by *flaA*-RFLP as described above.

### DNA sequencing of the entire *flaA* gene

The *flaA* amplicons of all isolates with a length of approximately 1.7 kb were excised from the gel and DNA was purified using the QIAamp Gel Extraction Kit (Qiagen, Hilden, Germany) according to the manufacturer's recommendations. Cycle sequencing was done with different sequencing primers (Table 2) using BigDye Terminator v1.1 Cycle Sequencing Kit (Applied Biosystems, Darmstadt, Germany) according to the recommendations of the manufacturer. Sequencing products were analyzed with a Genetic Analyzer ABI PRISM 3130 (Applied Biosystems). Whole *flaA* gene and short variable region (SVR) sequences were analyzed to identify the most parsimonious relationships. Based upon sequence data (accession numbers in Table 1) of investigated isolates a split network tree was constructed with cluster tree neighbour-joining analysis using the bioinformatics tools of Geneious V5.1 analysis [30]. Dendrograms were generated for both the entire *flaA* gene sequence and the *flaA*-SVR sequence (bases 450 through 600). This approach is compatible with the sequence typing and schemes used in the PubMLST database (http://www.pubmlst.org/). The *flaA*-SVR alleles (321 nucleotides from position 280 to 600) and peptides (n = 107) were calculated using the database available at (http://pubmlst.org/campylobacter).

### Multilocus sequence typing (MLST)

The MLST protocol was carried out as described previously [18]. The target fragments of the housekeeping genes were *aspA* (aspartase), *glnA* (glutamine synthetase), *gltA* (citrate synthase), *glyA* (serine hydroxyl methyl transferase), *pgm* (phosphor glucomutase), *tkt* (transketolase), and *uncA* (ATP synthase alpha subunit). PCR products were amplified with designed oligonucleotide primer pairs (Table 2). The reaction conditions were: denaturation at 94°C for 120 s, primer annealing at 50°C for 60 s, and extension at 72°C for 60 s for 35 cycles. Amplicons were examined via gel electrophoresis and purified using the QIAamp Gel Extraction Kit (Qiagen, Hilden, Germany) according to the manufacturer's recommendations. Cycle sequencing and analysis of sequencing products were done as described above. Alleles, sequence types (STs) and clonal complexes (CCs) were assigned and putative phylogenetic relationships among the STs were presented using a minimum spanning tree, which was constructed using the MLST database available at (http://pubmlst.org/campylobacter).

### Microarray DNA hybridization assay

The microarray typing was carried out based on gene loci which are absent or present in *C. jejuni* isolates using the ArrayTube^TM^ platform (Alere Technologies GmbH, Jena, Germany). Two types of AT^TM^ microarrays with spotted probes were used to differentiate *C. jejuni* isolates: *C. jejuni*-1 and Campy-2. The basic AT^TM^ sample processing and data analysis workflow were done using special commercial kits (Alere Technologies GmbH) according to the manufacturer's instructions (www.alere-technologies.com). Briefly, 1 µg of RNA free genomic DNA was amplified by a duplex PCR using random primers and biotin-16-dUTP to label the amplicons. The amplified labeled DNA was hybridized to both arrays for one hour under agitation at 45°C, washed, and quantified after a colorimetric reaction using horseradish peroxidase and TrueBlue substrate.

### Algorithm for the interpretation of array data

Hybridization signals were measured after 5 min precipitation using an ArrayTube transmission reader ATR-03 (Alere Technologies GmbH). Signals were processed using the Iconoclust software, version 2.3 in combination with the Partisan Array LIMS system which provided the specific algorithms. The photograph and raw signal intensity data were transferred to the Array Tube Analyzer software. Normalised intensities of the spots were calculated. The local backgrounds as well as spot intensities were measured, using only valid pixels within the automatically recognized spot area for the latter. Normalized intensities of spot values were calculated according to the following equation: 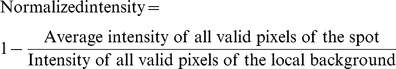



Numerical data were translated into logical data using cut-off values. Resulting values below 0.1 were considered negative (−) and those above 0.3 were considered positive (+), values between 0.1and 0.3 were regarded as ambiguous (+/−). For further analysis, the average of all probes for a given target allele was used. Cut-off values were defined based on the average normalized intensities of biotin staining controls and the hybridization controls [8].

The clustering of isolates was done based on the binary microarray data using the simple matching distance metric and UPGMA method of clustering in http://genomes.urv.cat/UPGMA/index.php?entrada using 100% fingerprint similarity for cluster definition. To estimate the strain relationships at a slightly lower level of discrimination, data were also analyzed at the 90% fingerprint similarity level, using DendroUPGMA, a dendrogram construction utility which creates a dendrogram without recalculation from a set of variables organizing all types related at ≥90% into single clusters [19].

### Evaluation of typing methods

Using the online tool for quantitative assessment of classification agreement (http://darwin.phyloviz.net/ComparingPartitions/index.php?link=Tool), the discriminatory power can be expressed by a numerical index of discrimination (D) as shown previously [27]. D value of 1.0 would indicate that a typing method was able to distinguish each member of a strain population from all other members of that population. Conversely, an index of 0.0 would indicate that all members of a strain population were of an identical type.

## Results

All fourteen isolates were confirmed as *C. jejuni* by Gram staining, biochemical tests, and multiplex PCR. The *flaA* gene was amplified using a modified PCR primer system (Table 2). It resulted in approximately 1.7 kb amplicons for all isolates.

**Figure 1:**
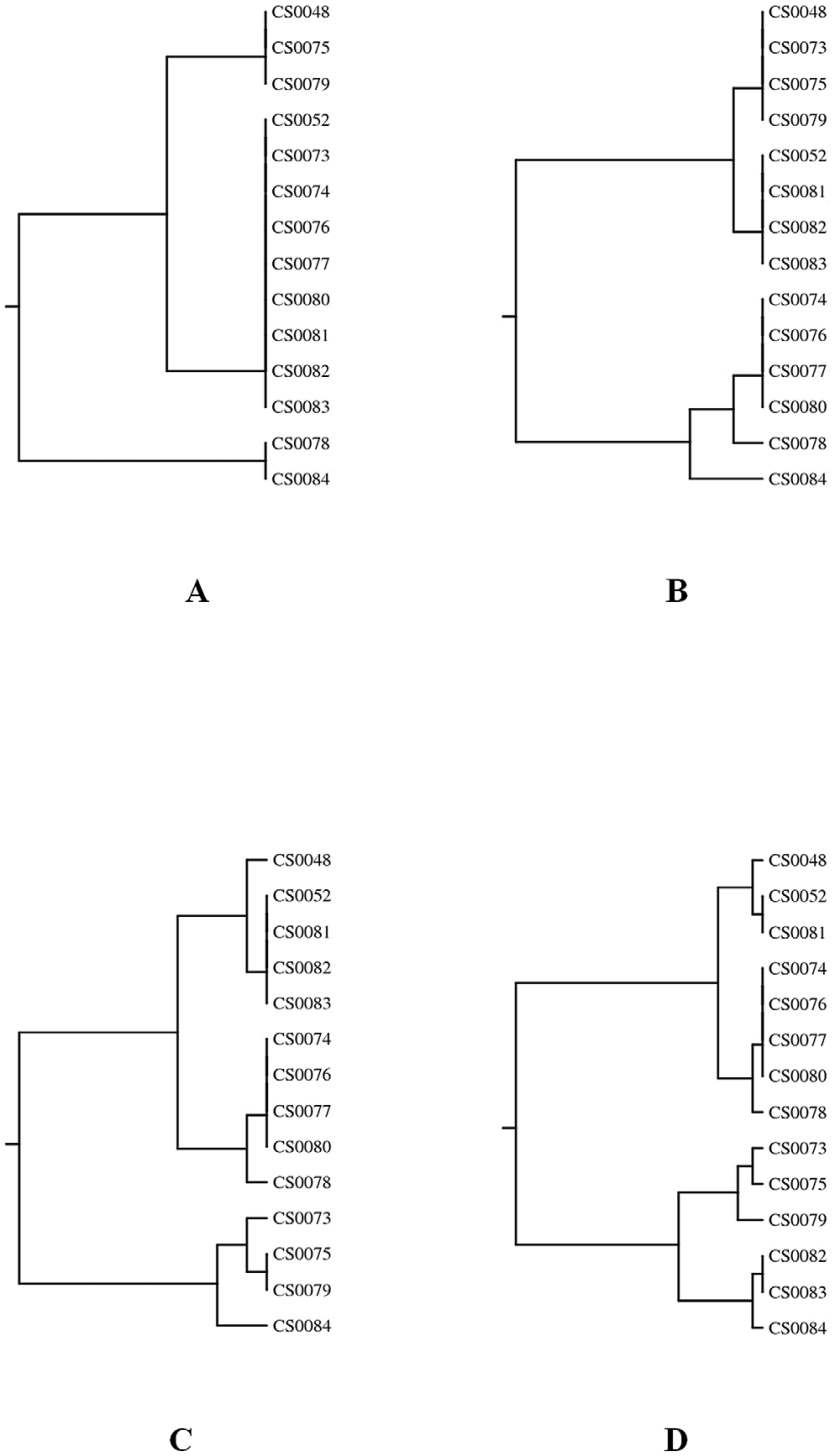
Dendograms based on restriction profiles of 14 *C. jejuni* isolates were digested using *Sau3AI* (A), *AluI* (B), a combination of *Sau3AI* and *AluI* (C), and *DdeI* (D). *flaA*-RFLP cluster analysis was performed with the Dice correlation coefficient and the unweighted pair group mathematical average clustering algorithm of BioNumerics ver. 4.50.

**Figure 2:**
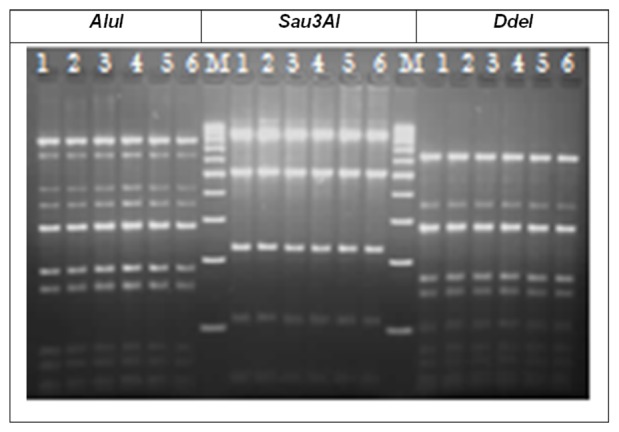
Agarose gel electrophoresis of PCR-RFLP profiles of *flaA* genes of *C. jejuni* isolate CS0078. Genetic stability was tested using *AluI*, *Sau3AI* and *DdeI*. Lane M: 100 bp ladder (Jena Bioscience GmbH), lane 1: *flaA*-RFLP patterns before *in vitro* passage, lane 2 to lane 6: *flaA*-RFLP patterns after 4^th^, 8^th^, 12^th^, 16^th^ and 20^th^ passages, respectively.

The PCR-RFLP analysis of *flaA* genes of the 14 *C. jejuni* isolates revealed 3, 5, 7 and 9 genotypes when digested with *Sau3AI*, *AluI*, and a combination of *Sau3AI* and *AluI*, and *DdeI*, respectively (Table 1 and Figure 1). All isolates gave identical results when experiments were repeated (data not shown). *In vitro* stress tests demonstrated genetic stability of *flaA* genes in seven selected isolates over 20 subcultivations (Figure 2).

**Figure 3:**
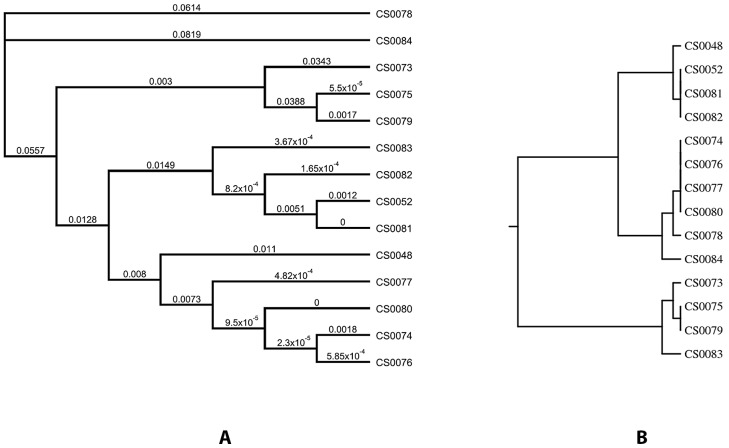
Relationships within 14 *C. jejuni* isolates based on entire *flaA* sequences and *flaA*-SVR sequences. The generated phylogenetic tree of the entire *fla*A sequences had 11 terminal taxa (A), while the results of *flaA*-SVR sequence typing generated 8 different types (B). Dendrograms were generated using CLUSTREE neighbour-joining analysis. Scale bar: 0.02 divergent residues per site. Congruent topologies (P<25%) obtained using Geneious V5.1 (13).

DNA sequences of entire *flaA* genes of these *C. jejuni* isolates (sequences are available in GenBank with the accession numbers given in Table 1) were grouped into a single alignment and were analyzed for the most parsimonious relationships. The generated phylogenetic tree (Figure 3) had 11 terminal taxa, thereby each taxon was assigned by a numerical designation representing a unique sequence. The *flaA* sequences are characterized by a higher level of variability between sequence positions 700 and 1450.

The results of *flaA*-SVR sequence typing of the 14 *C. jejuni* isolates are given in Table 1. Eight *flaA* alleles and 4 *flaA* peptides were detected. One new *flaA* allele type (528) was identified. The *flaA*-SVR dendrogram demonstrates a higher homology within these isolates than that obtained for the entire *flaA* genes (Figure 3).

**Figure 4:**
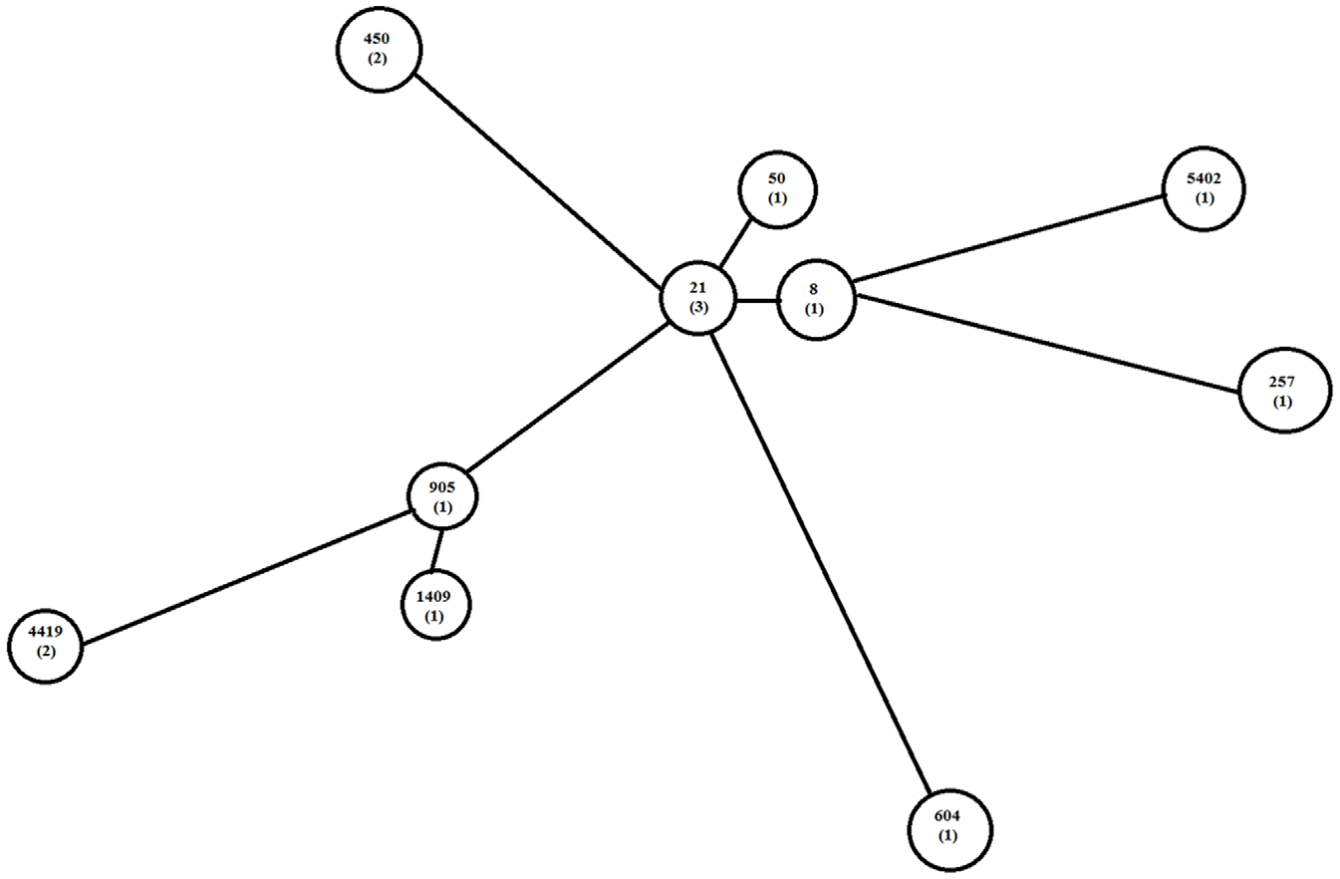
Minimum spanning tree depicting the clustering of 10 STs identified among 14 *C. jejuni* isolates. The tree was created using BioNumerics (version 4.6; Applied Maths). Each ST is represented by a circle. Numbers in brackets expressed numbers of isolates within a ST. The ST designations were obtained from http://pubmlst.org/campylobacter.

MLST analysis identified 10 STs, 7 of which (50, 5402, 604, 8, 905, 1409, 257) were present only once (Table 3). The minimum spanning tree was constructed to show the relatedness among the 10 STs (Figure 4).

**Table 3:** Allelic profiles and resulting sequence types (STs) and clonal complexes (CCs) for 14 *C. jejuni* isolates by using MLST analysis.

Isolate	Allelic profile^A^							ST	CC
	*aspA*	*glnA*	*gltA*	*glyA*	*pgm*	*tkt*	*uncA*		
CS0048	2	1	12	3	2	1	5	50	ST-21
CS0052	2	115	298	26	127	29	35	4419	not found
CS0073	8	10	2	2	2	2	6	5402	ST-354
CS0074	2	1	1	3	2	1	5	21	ST-21
CS0075	47	55	5	10	11	48	8	450	ST-446
CS0076	2	1	1	3	2	1	5	21	ST-21
CS0077	2	1	1	3	2	1	5	21	ST-21
CS0078	1	2	3	27	5	9	3	604	ST-42
CS0079	47	55	5	10	11	48	8	450	ST-446
CS0080	2	1	1	3	2	1	6	8	ST-21
CS0081	2	115	298	26	417	29	35	4419	not found
CS0082	2	15	4	3	154	25	35	905	not found
CS0083	2	15	4	3	154	51	35	1409	not found
CS0084	9	2	4	62	4	5	6	257	ST-257

A http://pubmlst.org/campylobacter.

The dynamics of the colonization of the turkey flock by different *C. jejuni* isolates during the rearing process could be demonstrated (Table 2). The first *C. jejuni* ST 50 represented by CS0048 was substituted with other genotypes over a period of 11 weeks and could never be re-isolated. On the other hand, ST 4419 (CS0052) was isolated again after 5 weeks of the first finding (CS0081). At date 07.07.2010, 9 isolates were recovered which represented 5 coexisting STs. No dominating ST was found alongside the production process.

**Figure 5:**
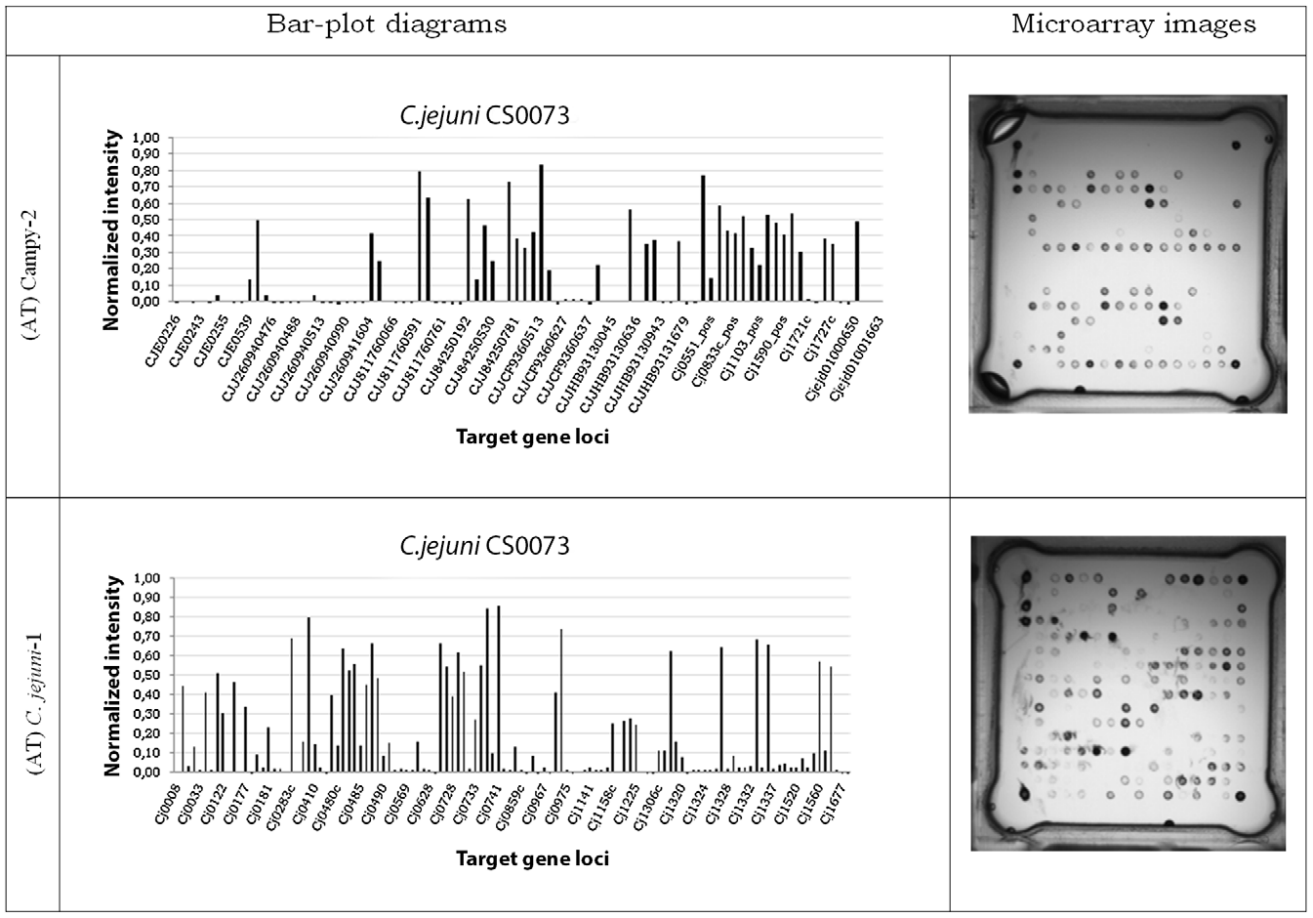
Hybridization patterns for *C. jejuni* isolate CS0073 presented as microarray images and bar-plot diagrams. The tested gene loci were arranged on two chips: *C. jejuni*-1 and Campy-2. The normalized intensity signal >0.3 considered positive.

**Figure 6:**
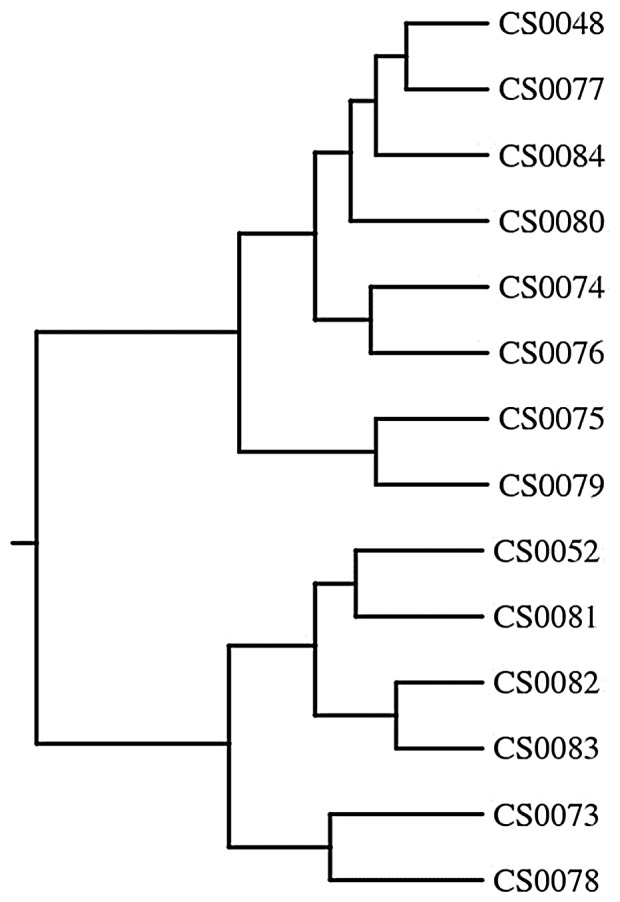
Dendrogram based on microarray data using DendroUPGMA. The clustering of *C. jejuni* isolates represents 14 different clusters based on the binary microarray data using the simple matching distance metric and (UPGMA) using average linkages.

The microarray analysis showed a high level of discrimination (1.00) between *C. jejuni* isolates based upon different gene targets as shown in Figure 5. The microarray demonstrated as images and bar plot diagrams. The signal intensities express the absence or presence of different gene loci in the genome of *C. jejuni* isolates. The analysis of the hybridization images using the simple matching distance metric and UPGMA resulted in 14 different clusters. The dendrogram (Figure 6) illustrates the relatedness of the isolates based upon hybridization data.

The performance of the different genotyping systems was assessed based upon the index of discrimination, the costs per isolate, ease of handling, and time-to-result for one sample as shown in Table 4. Briefly, the highest discriminatory index was achieved using the microarray method (1.00). The entire *flaA* sequence analysis represented a higher discrimination (0.967) than MLST analysis (0.945) and *flaA*-SVR sequence analysis (0.890). The calculated D indices for the PCR-RFLP of the *flaA* genes with *DdeI* were higher (0.9121) than those for *Sau3AI, AluI* and the composite of *Sau3AI* and *AluI* digestion (0.5604, 0.8022, and 0.8571), respectively.

**Table 4:** Comparison of the performance of *flaA* typing, MLST analysis and DNA microarray assay (ArrayTube^TM^ technology) of 14 *C. jejuni* isolates.

Typing technique	types	discriminatory Index D A	CI (95%) A	CINA (95%) A	Time (h)	Costs/sample (€)	Equipment
***flaA*** ** PCR-RFLP**							PCR thermocycler, Electrophoresis, Incubator
(a) *Sau3*AI	3	0.560	(0.325–0.796)	(0.311–0.810)	18	3,00	
(b) *Alu*I	5	0.802	(0.722–0.882)	(0.683–0.922)	18	3,00	
(c) *Sau3*AI and *Alu*I	7	0.857	(0.754–0.961)	(0.727–0.988)	18	3,00	
(d) *Dde*I	9	0.912	(0.817–1.000)	(0.794–1.000)	18	3,00	
**Entire ** ***flaA*** ** sequencing**	11	0.967	(0.929–1.000)	(0.894–1.000)	10	24,00	PCR thermocycler, Electrophoresis
***flaA*** **-SVR sequencing**	8	0.890	(0.796–0.985)	(0.770–1.000)	10	15,00	PCR thermocycler, Electrophoresis
**MLST analysis**	10	0.945	(0.884–1.000)	(0.855–1.000)	12	70,00	PCR thermocycler, Electrophoresis, Genetic analyzer
**DNA microarray**	14	1.000	(1.000–1.000)	(0.946–1.000)	5	30,00	PCR thermocycler, Thermomixer, ArrayTubes (AT™), ArrayTube Reader

A The online tool at the Comparing Partitions website (http://www.comparingpartitions.info/) was used for this analysis. CI (95% confidence interval); CINA (95% non-approximated confidence interval).

The presented results could be obtained in 5 hours for the microarray assay, 10 hours for DNA sequencing, more than 18 hours for PCR-RFLP and 12 hours for the MLST assay. The PCR-RFLP method was found to be a cheap method for typing *Campylobacter* with a cost of 3.00 € per isolate, while the corresponding costs for entire *flaA* gene sequencing, MLST and the microarray analysis reached 24.00 €, 70.00 € and 30.00 €, respectively.

## Discussion

Thermophilic *Campylobacter* continues to significantly contribute to the worldwide public health impact. Understanding the epidemiology of *Campylobacter* spp. can help to reduce the disease burden.

The genetic diversity amongst *Campylobacter* must be considered in epidemiological evaluations and microbial risk assessments of *Campylobacter* in poultry. Multiple genotypes can constitute the *Campylobacter* population within poultry flocks, suggesting different sources of exposure and/or genetic drifts within the *Campylobacter* population [4].

This study aimed to elucidate the genetic diversity among 14 *C. jejuni* isolates recovered from a single commercially reared turkey flock. These isolates were sampled from the beginning of the colonization until the slaughtering of the turkeys. Molecular biological typing was done using established *flaA* typing methods, MLST, and DNA microarray assay based on the ArrayTube^TM^ technology. The usefulness of the different typing systems was evaluated.

The present study demonstrated that a single turkey flock can be simultaneously colonized with more than one *Campylobacter* genotype during the rearing cycle. It also reflected the changing in the occurrence of different types of *C. jejuni* between age 4 to 15 weeks. Types which were originally observed were substituted by others and could not be re-isolated anymore. Specific genotypes were also repeatedly identified at different rearing moments. Furthermore, investigations showed the simultaneous coexistence of different types at a single date. The dominant *C. jejuni* type could not be found in this turkey flock. No genotype was found which was present over the whole investigation period.

The situation in chickens was similarly described. Broiler flocks were identified in which different *Campylobacter* clones coexisted [4]. In contrast, other studies [31], [32] reported the detection of only one genotype per sampled flock.

Molecular methods used for typing of *C. jejuni*, which are characterized by low complexity and high reproducibility, are needed to study the bacterial population structure.

The use of *flaA* gene typing for epidemiological studies is controversial, due to the intra- and inter-genomic recombination within the flagellin genes that results in significant sequence heterogeneity [33]. PCR primers previously used for *flaA* amplification were found to be insufficient to amplify this gene in certain isolates [6]. Here, “wobbled” primers were used to amplify *flaA* genes. The discriminatory power of *flaA*-RFLP typing clearly depended upon the type of restriction enzyme used [9], [33]. The results showed that cluster analysis based on composite digestion (*AluI* and *Sau3AI*) of *flaA* genes was more discriminative than either single (*AluI* or *Sau3AI*) enzyme *flaA* typing. Moreover, the highest discriminatory power in *flaA*-RFLP typing was achieved using *DdeI*. It was confirmed that *flaA*-RFLP typing should not be used alone to genotype the isolates [11], due to the dependence of the results on the enzyme used and the limitation of the *flaA* gene being a very small part of the whole genome. PCR-RFLP assays are difficult to standardize and inter-laboratory comparisons of results are often ambiguous.

DNA sequencing of entire *flaA* genes resulted in greater discriminatory power (D = 0.967) than that obtained with PCR-RFLP methods. DNA sequencing is done routinely in many research laboratories or is available as a low cost commercial service and the results can easily be exchanged among laboratories [12], [13], [15]. In contrast to a previous report [12], this study demonstrated that the genetic relatedness derived from the *flaA*-SVR sequence did not correlate with that obtained by the entire *flaA* gene sequence. The D index of *flaA*-SVR analysis was lower (D = 0.890) than that obtained by entire *flaA* gene sequencing (D = 0.967).

MLST is an important technique that provides a reliable prediction of clonality for population studies of *Campylobacter* spp. with high discrimination [18], [19], [33]. Furthermore, another benefit of MLST is that assignment of DNA sequences to MLST alleles and sequence types is not prone to the variation and interpretation of restriction band profiling and band migration through the electrophoretic medium and MLST sequence data can be readily compared between laboratories [19].

In this study, the MLST analysis represented 10 different sequence types and 5 clonal complexes. Two identified STs (ST 450 and ST 257) were previously reported in turkey. Other STs (50, 4419, 21, 604, 905 and 1409) were observed from chicken and from human cases of illness; ST 5402 was detected in pork offal and ST 8 was detected in cattle and sheep (http://www.pubmlst.org/campylobacter). Although MLST results are easy to reproduce, interpret and transfer, it is a complex, labor-intensive and expensive technique to perform in comparison with other typing methods used in this study as well as described earlier [34].

The ArrayTube^TM^ microarray system is also relatively inexpensive, when hands-on-time, necessary equipment, and time are considered. It can be carried out automatically, as it is based on a simple spot pattern recognition assay and had very high throughput and a short turnaround time when compared to the other molecular typing methods assessed here.

The multistep method, DNA microarray analysis (includes amplification, labeling, hybridization etc.) is not technically difficult. The results of the analysis are given as images and bar-plot diagrams where positive and negative hybridization signals are differentiated at a value of 0.3 (Figure 5). Data analysis of microarray results is a simple computerized step. The whole procedure using the AT^TM^ system is an extremely portable process which needs only a minimum of standardization [25], [35]. A further benefit of the microarray assay is the integration of the whole genome in the investigation instead of only one or a limited number of genes.

In general, a method that yields discrimination values of higher than 0.95 can be considered more or less “ideal” [11]. However, selection of the typing method depends upon many variables such as cost, difficulty of technique performance, and interpretation of results.

On the basis of discriminatory power, DNA microarrays (D = 1.00) appear to be the preferred method used for typing of *C. jejuni* through routine surveillance.

In summary, it was shown that different typing methods reveal the same results: a genetic heterogeneity of *Campylobacter* isolates from turkey during the rearing process was observed, but the degree of relatedness was different depending upon the typing method. Use of more than a single method gives clarity about the genetic heterogeneity within the *Campylobacter* population.

The *C. jejuni* isolates were shown to be genetically stable during 20 *in vitro* passages corresponding with findings of others [12], [36], [37], [38]. However, previous reports also described genomic instability among campylobacters [9], [39], [40].

In the present study, the genetic diversity among *C. jejuni* isolates was investigated in a single turkey flock. The turkeys harbored more than one genotype of *C. jejuni* in the same rearing cycle. The investigation demonstrated clearly a dynamic in changing of the *Campylobacter* population in turkeys as well as a coexistence of different genotypes for the first time, to our knowledge, during turkey rearing. The heterogeneity profiles differed according to the typing methods in which DNA microarray-based comparative genomic hybridization analysis provides the most powerful alternative for *C. jejuni* genotyping.

Based upon the results of genotyping and *in vitro* stability tests, it seems clear that different strains had colonized the turkeys at different dates, alongside the rearing process. The sources for *Campylobacter* transmission into the turkey flock are not yet known.
